# Migraines, Obesity, and Pregnancy: Who Is the Villain and Who Is the Victim?

**DOI:** 10.3390/life15071014

**Published:** 2025-06-25

**Authors:** Milan Lackovic, Sladjana Mihajlovic, Jovana Kuzmanovic Pficer, Ivan Hristov, Zagorka Milovanovic, Ivona Jovanovic, Dejan Nikolic

**Affiliations:** 1 Harris Birthright Research Centre for Fetal Medicine, King’s College Hospital, London SE5 8BB, UK; milan.lackovic@nhs.net; 2 Department of Obstetrics and Gynecology, University Hospital “Dragisa Misovic”, 11000 Belgrade, Serbia; drsladjanamihajlovic@gmail.com (S.M.); hristov.ivan@outlook.com (I.H.); 3 Faculty of Medicine, University of Belgrade, 11000 Belgrade, Serbia; zaga_mil@yahoo.co.uk; 4 Department of Medical Statistics and Informatics, Faculty of Dentistry, University of Belgrade, 11000 Belgrade, Serbia; jovana57@yahoo.com; 5 Clinic for Gynecology and Obstetrics “Narodni Front”, 11000 Belgrade, Serbia; ivona.vuceljic@gmail.com; 6 Department of Physical Medicine and Rehabilitation, University Children’s Hospital, 11000 Belgrade, Serbia

**Keywords:** migraine, pregnancy, obesity, gestational diabetes, comorbidities

## Abstract

Introduction: Migraines are a prevalent neurovascular disorder that affects more than a billion people worldwide. Even though both women and men are affected by this neurological disorder, migraines are primarily recognized as a women’s health disruption factor. Pregnancy leads to significant hormonal changes, including a rise in estrogen, progesterone, and endogeny opioid levels, and, therefore, it can affect the course of migraines. Women dealing with migraines often experience migraine symptom reduction during the course of pregnancy, but in the setting of increased maternal body mass index and obesity, this common pattern may be altered. Due to the complexity of the interplay between pregnancy, obesity, and migraines, all mediated by hormonal changes, the aim of our study is to try to unravel the impact of migraines and obesity on maternal health and pregnancy outcomes. Methods: This study included 350 subjects who have suffered at least one migraine attack three months preceding pregnancy, or at any point during the course of pregnancy. Initially, the study subjects were divided into two groups. The first group included women suffering from migraines before pregnancy, but not during the course of pregnancy, and the other group included all subjects who remained symptomatic during the course of pregnancy or had a first migraine attack during their pregnancy. Further comparisons were made based on the patients’ BMI values, and correlations were made between the obese and non-obese study subjects. Results: Higher parity (*p* = 0.005), obesity (*p* = 0.005), earlier age of migraine onset (*p* = 0.004), and gestational diabetes mellitus (*p* = 0.004) were statistically significant predictors for migraine symptom persistence during pregnancy. Obese pregnant women were more likely to experience migraine intensity and frequency persistence during pregnancy (*p* < 0.001 and *p* < 0.001, respectively). They sought magnesium treatment more often (*p* < 0.001), but this had a modest therapeutic effect compared to non-obese pregnant women (*p* < 0.001). A diagnosis of hypertensive disorder of pregnancy and gestational diabetes mellitus was also more frequently established in the group of obese pregnant women (*p* = 0.002 and *p* < 0.001, respectively). Conclusions: Pregnancy induces substantial physiological changes that can both alleviate and exacerbate migraine symptoms. Obesity is a modifiable risk factor that not only may increase the intensity and frequency of migraine symptoms, but may also compromise pregnancy course and outcome. The co-occurrence of migraines and obesity during pregnancy may amplify health risks for the mother and fetus, including heightened susceptibility to gestational diabetes mellitus. Future research should prioritize focusing on better understanding the causal relationships between pregnancy, migraines, and obesity and providing treatment strategies the home in on weight management and the control of migraine symptoms and associated comorbidities.

## 1. Introduction

Migraines are a prevalent neurovascular disorder that affects more than a billion women and men worldwide. They are characterized by recurrent, usually severe headaches that are often accompanied by nausea, vomiting, sound and light sensitivity, and associated disability [1]. Despite both women and men being affected by this neurological disorder in a similar frequency pattern, there is a notable discrepancy in migraine prevalence, distribution, duration, and disability between the genders. Contemporary epidemiological data have undoubtedly supported findings that migraines are primarily recognized as a women’s health disruption factor [2].

One of the most commonly used mechanisms for explaining the gender imbalance in migraine manifestations relies on hormonal differences displayed between the genders and the implication of hormonal changes on neuronal function, as well as the reactivity of vascular structures. Sex steroids cross the blood–brain barrier and are present in similar concentrations in cerebral and systemic circulation. Estrogen and progesterone hormonal shifts affect multiple central nervous system (CNS) regions involved in the pathophysiology of migraines and play a prominent role in vascular tone control and modulation. The regulation and modulation of CNS excitability by sex steroids is due to the neuron excitability increase accompanied by the acute sex steroid exposure, ion channel alterations, and ligand–receptor interaction alteration [3]. Furthermore, their course of action is immediately dependent on the activation of estrogen and progesterone receptors involved in the metabolism and production of the neurotransmitters and hormones in the CNS, including serotonin, noradrenalin, glutamate, nitric oxide, calcitonin gene-related peptide, and endogenous opioids [4]. This complex interplay of sex steroids within the CNS structures and the magnitude of estrogen withdrawal impact migraine symptom manifestations, including those of menstrual migraines, which has confirmed that migraine attacks which are associated with menstrual cycle are associated with significantly greater disability compared to attacks that are not associated with menstrual period and sex steroid changes [5]. Aside from their CNS manifestations, the action of sex hormones is tightly connected to vascular tone modulation, and it is known that, by regulating vascular tone inhibition through the action of estrogen, women have a more favorable cardiovascular system performance compered to men [6].

Obesity is associated with several health conditions resulting from chronic inflammation and hormonal disruption, including metabolic disorders and cardiovascular disease, which are known to potentially influence migraine pathophysiology. Obesity is identified as a risk factor for a deteriorating severity and intensity of migraine attacks, but the causative pathophysiology mechanism linking it to migraines still remains unclear [7]. Reproductive age, age of the most turbulent hormonal changes, is identified as the most relevant age when it comes to the interplay of the pathophysiological mechanisms connecting migraines and obesity, while, after the age of 55, obesity appears not to have any role in migraines symptom display at all [8]. Aside from being a risk factor for maternal health and the pregnancy course, obesity and associated complications have short-and long-term implications regarding the wellbeing of the offspring [9,10].

Pregnancy leads to significant hormonal changes, including a rise in estrogen, progesterone, and endogeny opioid levels, and, therefore, it can affect the course of migraine symptoms [11]. Women dealing with migraines often experience migraine symptom reduction during the course of pregnancy, especially during the second and third trimester of pregnancy [12]. However, in the setting of increased maternal body mass index (BMI) and obesity, this common pattern may be altered. The migraine phenotype was recently proposed as an integral part of perinatal risk factors assessment, which led to the search for a more proactive course of action, with blood thinners even being suggested as a method of prevention and treatment for individuals caring a burden of a migraine history [13].

Migraines during pregnancy may present an isolated risk factor for maternal health and the course of pregnancy, but there is also a possibility that they might have an unfavorable synergistic effect with obesity during pregnancy, which is commonly associated with other pregnancy-related complications and conditions. Therefore, it is relevant to assess the impact of these two prevalent disorders that affect the vulnerable population of pregnant women. Due to the complexity of the interplay between pregnancy, obesity, and migraines, all mediated by hormonal changes, further research focusing on the pregnancy outcomes in this vulnerable group of patients remains critical for improving perinatal care and women’s health overall. Hence, the aim of this study is to try to unravel the mutual interferences that exist between migraines and obesity during the course of pregnancy.

## 2. Methods

### 2.1. Study Design and Participants

This clinical observational study included 350 subjects who had regular pregnancy monitoring and gave birth in the University Hospital “Dragisa Misovic” in Belgrade, Serbia. Principles of the Declaration of Helsinki and good clinical practice were applied, and this study obtained approval from the Institutional Review Board (IRB) (No. 11297/3, Date: 27 May 2024).

### 2.2. Study Participants

Enrollment of the study participants was initiated during the first trimester of pregnancy (gestational weeks range 7–11) and was carried out throughout the entire course of pregnancy. This study included all pregnant women who have suffered at least one migraine attack three months preceding pregnancy, or at any point during the course of pregnancy. Diagnosis of migraine was made according to diagnostic criteria proposed by the classification of the International Headache Society (IHC) [14]. Due to the subjective nature of the symptoms, possible overlap with other causes of headache, and variability between individuals, it is often challenging to standardize migraine diagnosis. Therefore, enrolled study subjects underwent a neurological examination that ruled out secondary causes of headaches, and clinical diagnosis based on IHC criteria was established. All study subjects filled out a detailed questionnaire on their first perinatal visit as well as three months following the delivery, and the remaining clinical data were collected from the patients’ medical history. Prior to inclusion in the study, a written consent was obtained, and all study subjects consented to participating in the study.

Initially, all study subjects were divided into two study groups based on the records of their migraine symptom manifestations during the course of pregnancy. The first group included women suffering from migraines before pregnancy, but not during the course of pregnancy, and the other group included all subjects who remained symptomatic during the course of pregnancy or had a first migraine attack during pregnancy. Further comparisons were made based on the participants’ BMI values, which were measured at the time of the first perinatal visit. Subjects were divided into two groups, obese and non-obese study subjects, and comparisons of migraine symptoms were made. A BMI value higher than 30 kg/m^2^ was taken as the cut-off value.

### 2.3. Exclusion Criteria

Women aged less than 18 years old or older than 45 years old were excluded from this study, and so were women with chronic health conditions, aside from migraines, and multiple pregnancies.

### 2.4. Study Variables

#### 2.4.1. Maternal Variables and Migraine Characteristics

Maternal obesity (BMI > 30 kg/m^2^); parity; thyroid gland disfunction (hypothyroidism or hyperthyroidism); age of life when the migraines first occurred (migraine onset); family history of migraines in the first degree; whether or not migraines are related to menstrual cycles; whether or not they are they are triggered by daily stress, sleep deprivation, or weather changes; migraine pain intensity during pregnancy versus pre-pregnancy; frequency of migraine attacks during pregnancy versus pre-pregnancy; migraine pain intensity postpartum versus during pregnancy; frequency of migraine attacks postpartum versus during pregnancy; migraine frequency during the three trimesters of pregnancy; magnesium treatment; results following magnesium treatment (patients who used this type of treatment reported no change of symptoms or quick resolution of symptoms) were all considered. Magnesium at 350–400 mg/day was prescribed by a neurologist as an oral suspension, and none of the study subjects experienced any sign of magnesium toxicity.

#### 2.4.2. Perinatal Variables

Prematurity (gestational age less than 37 weeks), fetal macrosomia, intrauterine growth restriction (IUGR), delivery mode (vaginal delivery, Cesarean section, assisted delivery), pregnancy related hypertension (PRH), and gestational diabetes mellitus (GDM) were considered as perinatal variables. Fetal macrosomia was defined as newborn’s birth weigh more than 4000 g [15], and IUGR was defined according to Delphi consensus [16]. PRH was defined as blood pressure above 140/90 mmHg, and diagnosis of GDM was established according to American Diabetes Association (ADA) criteria [17]. Patients diagnosed with GDM were further subcategorized into two groups. GDM group 1 patients required medicamentous treatment (metformin or insulin, or the combination of the two), and GDM group 2 patients were only diet controlled.

### 2.5. Statistical Analysis

Analyses were done in the IBM SPSS statistical software package for Windows, Version 22.0. (Released 2013. Armonk, NY, USA: IBM Corp.). Categorical data are described as frequencies and percentages, while numerical data are presented as means and standard deviations. The Chi-square test (χ^2^) was used for categorical data analysis. The distribution of continuous numerical data was evaluated using the Kolmogorov–Smirnov test. The Mann–Whitney U test was employed to statistically assess differences within the non-parametric data. To identify predictive factors that influence the presence of migraine, univariate and multiple logistic regression analyses were performed.

## 3. Results

This study included 350 women diagnosed with migraines. The study subjects had migraines either only prior to pregnancy, or the migraine symptoms persisted during the entire course of pregnancy. Ninety-five study subjects reported complete migraine symptom remission during pregnancy, while the remaining 255 subjects remained symptomatic or experienced their very first migraine attack during the pregnancy. Table 1 presents the distribution of the evaluated study variables which are related to the presence of migraine symptoms during pregnancy. Pregnant women who remained symptomatic during pregnancy had higher parity (*p* = 0.039), they were more commonly obese (*p* < 0.001), and GDM was more frequently diagnosed (*p* < 0.001).

Out of 350 enrolled study subjects, 100 were obese. Table 2 presents the distribution of evaluated variables related to the observed body mass index values. Obese subjects (BMI > 30 kg/m^2^) were significantly more likely to have hypertensive disorder of pregnancy (*p* = 0.002), gestational diabetes mellitus (*p* < 0.001), and their migraine intensity and frequency persist during the pregnancy (*p* < 0.001 and *p* < 0.001, respectively). During the first and the third trimesters of pregnancy, there was a notable difference in migraine symptom frequency between the two compared groups. Furthermore, obese subjects were more likely to seek additional treatment options, such as magnesium treatment (*p* < 0.001), but unfortunately more than half of the patients who were obese did not report any change in symptom control after magnesium treatment, unlike the group of patients who were non-obese, in which quick symptom resolution was more frequently observed (*p* < 0.001).

Table 3 shows that the statistically significant predictors regarding the presence of migraine during pregnancy were parity (OR: 1.766; 95%CI: 1.190–2.619; *p* = 0.005); the BMI category (OR: 2.691; 95%CI: 1.347–5.376; *p* = 0.005); the age of migraine onset (OR: 0.918; 95%CI: 0.865–0.974; *p* = 0.004); and gestational diabetes mellitus (OR: 4.788; 95%CI: 1.915–11.970; *p* = 0.001). The results show that there is a 1.77 times greater chance of migraine persistence during pregnancy with an increase in the number of deliveries; obese women had nearly a 2.7 times greater chance of having migraine symptoms during pregnancy, while the earlier occurrence of migraines in life indicates a greater chance of migraines in pregnancy. Also, the probability of migraine in pregnant women with diet-controlled GDM increased by about 4.79 times compared to women without diabetes.

## 4. Discussion

Our study included 350 women diagnosed with migraines, who were initially divided into two groups based on their migraine symptom manifestation during the course of pregnancy. More than 70% of the enrolled subjects remained symptomatic during the pregnancy, while, in less than a third of subjects, migraine attacks completely ceased. According to the statistical analysis, the two main characteristics associated with migraine manifestation persistence were maternal obesity and gestational diabetes. The burden of obesity was observed nearly three times more frequently in the group of women who remained symptomatic during the course of pregnancy compared to the women who experienced symptom relief. Every third women suffering from migraine during pregnancy was obese, and the diagnosis of GDM was established with a similar percentage rate. To the contrary, among the women who had migraine symptom remission during pregnancy, GDM was diagnosed in less than 10% of cases, and only 12.6% of them were obese.

A review of the literature confirms that migraines and obesity share common pathophysiological mechanisms. By causing sympathetic nervous system dysregulation and hypothalamic dysfunction, chronic inflammation associated with both obesity and migraines leads to interceptions of pathophysiological mechanisms that predispose individuals to migraine symptom chronification [18]. Furthermore, migraine history is identified as a risk factor for a significant weight gain leading to obesity during the onset of reproductive age [19], a part of women’s life in which they are recognized as vulnerable due to the turbulent changes caused by sex hormones shifts. Pregnancy, known for the intense hormonal fluctuations needed to support uneventful pregnancy progression, but also for the susceptibility to weight gain of pregnant women [20,21], is therefore another vulnerable stage of women’s life in which the interaction of obesity and migraines may cause migraine symptom maintenance or even amplification, as well as obesity exacerbation. Fan et al. found a profound interrelation between migraines and obesity, indicating that not only does obesity affect migraine symptom pattern display, but that the obesity trajectory and tendency is also raised in patients with protracted migraine episodes [22]. Pre-pregnancy obesity is recognized as a risk factor for excessive gestational weigh gain, another pregnancy-related risk factor that presents an additional burden to the already-comprised maternal health and displays cumulative synergistic negative effects on the pregnancy outcome and the wellbeing of the offspring [23]. Therefore, the disturbed hormonal balance associated with maternal pre-pregnancy obesity might be further aggravated by gestational weight gain, which could lead to a failure of the migraine symptom release that is commonly associated with the normal course of pregnancy [24]. Altered levels of sex hormones during pregnancy in obese women have been described in a study published by Maliqueo et al., but due to the differences in placental aromatase expression in male and female fetuses, the authors stressed that is important to take into account that these changes are dependent on the fetal gender as well [25].

Moreover, pregnancy in itself and obesity share some other common characteristics. Weight gain and chronic inflammation, core features of obesity, are an integral part of the physiological process of maternal adaptation to pregnancy. Another remarkable similarity is the increased insulin resistance (IR) that is present in both conditions, pregnancy and obesity. Joined together, the two conditions have a synergistic effect that predisposes the mother to increased IR, more intense systemic inflammatory reaction, and metabolic and immune system changes, and thereby changes in the hormonal milieu as well [26,27]. 

Aside from being a common feature aligning obesity and migraines, the presence of IR is one of the pathophysiological hallmarks of the most prevalent endocrinological disorder of pregnancy, GDM [28]. Musa et al. found that not only do obesity and GDM often go hand in hand, but they also have a specific impact on placental function and hormone secretion. This research group has investigated possible effects of interactions between GDM and maternal obesity on placental morphology and hormone and inflammatory cytokine secretion, and found that, by modulating the placental morphology and development, GDM and obesity have a specific impact on maternal and fetal endocrine and inflammatory status that leads to unfavorable pregnancy outcomes [29]. 

Both migraines and gestational diabetes involve inflammatory markers and hormonal shifts, and during pregnancy, hormonal changes might exacerbate both conditions. Possible connections and interactions between diabetes and migraines have been explored in several studies, but meta-analyses that provide better insight into this important research topic are still lacking [30]. Migraines are a neurovascular disorder and have endothelial dysfunction as their vascular risk factor marker [31]. For a long time, it was common knowledge that vascular disease is a disease of older age, until it was disclosed that altered glucose metabolism leads to an early vascular aging and predisposes individuals to various cardiovascular conditions [32]. Obesity affects the vascular remodeling process in a similar fashion, leading to vascular disfunction and vascular disease [33]. Along with IR, this triad formed of vascular compromise components might be a linking mechanism that brings migraines, maternal obesity, and GDM into close association. Understanding the intriguing and powerful interrelations that connect the components of the vascular triad might provide deeper insight into the effects, as well as into the potency, that each of the components of the vascular triad have one on the other.

Hypertensive disorder of the pregnancy (HDP) is a manifestation resulting from dysfunctional systemic vascular resistance [34], and therefore it is not surprising that, in our study results, the distribution of HDP was significantly more prevalent in the group of patients who were obese who were dealing with migraines, who were at the same time more likely to have GDM. Despite usually being a transient finding and vanishing postpartum, HDP remains a life-long risk factor for cardiovascular disease (CVD), and as proposed by leading cardiologist associations as a result of the screening and follow-up of GDM, metabolic disorders and obesity after pregnancy accompanied by HDP should be a critical part of CVD prevention for this population of women [35]. Both HDP and migraines are known to increase CVD risk on their own, and it was recently confirmed that, for women dealing with both migraines and HDP, the risk of a premature major cardiovascular or cerebrovascular event is significantly increased [36]. Stroke is certainly one of the most severe complications following CVD, and unfortunately it may be a succeeder of both unregulated hypertension and uncontrolled migraines [37]. 

As we have previously discussed, metabolic changes and chronic inflammation affect patterns of hormone secretion and action [26,27], and, therefore, in the context of hormonal changes and shifts, we believe that another two parameters identified as relevant by our statistical analysis, age of migraine onset and parity, should not be left out or neglected from further discussion. Early migraine onset is associated with increased intensity and frequency of migraines, and migraines occurring at the younger age of life may also predict a longer course of disease [38,39]. We have found a significant difference between the age of migraine onset between the group of women who had and did not have migraines during pregnancy, since women who tended to have migraine symptom chronification throughout pregnancy were of a younger age when the initial migraine symptoms started. Even though pregnancy brings many joyful moments and benefits to women’s health, it undoubtedly affects cardiovascular system performance. Pregnancy-associated complications, such as GDM, HDP, or altered dynamics of fetal in utero growth, have a significant impact on the future CVD risk in women, and a history of multiple pregnancy-related complications further increases this risk [40]. It has been reported that parity plays an important role in CVD development, and a meta-analysis found that there is a risk linking the number of pregnancies and CVD risk [41]. The association found between a higher number of deliveries and migraine symptom manifestations might suggest that higher parity may lead to more prominent changes in vessels and possibly vascular disfunction, bringing us once again back to a vicious cycle connecting migraines, pregnancy, and obesity, all gathered around the phenomena of the vascular triad. Although some research groups have reported improvements in migraine symptom control following higher parity, attributing this mainly to cumulative hormonal changes during multiple pregnancies, others have reported no significant link, or even an increased risk of migraines postpartum [42,43]. The stress of parenthood, anxiety, and sleep deprivation might be a triggering factors for migraine manifestations, and, therefore, they may predispose individuals to displaying migraine symptoms. Nevertheless, pregnancy is one-of-a-kind stress test for the cardiovascular system [44], and in the setting of compromised vascular performances, predisposed by the vascular triad, it should not be surprising that physiological mechanisms that regulate cardiovascular and cerebrovascular performances might be struggling or even be led to a breaking point.

Tailoring optimal migraine treatment remains difficult and challenging, especially when it comes to pregnancy. It is known that, by reducing the risk factors that cause migraines, such as obesity and related comorbidities, optimal symptom control may be achieved. Physical activity during pregnancy has been proven to be beneficial for migraine symptom control [45], but since it requires significant compliance from patients, as well as an uneventful course of pregnancy, it is not always easily applicable in the setting of patients with a high BMI and associated comorbidities. While it balances between harmless and most effective, the largest number of our study patients opted for magnesium intake as a treatment option. For migraine symptom control, obese pregnant women opted for magnesium as a treatment option 50% more often compared to the group of women who were normal weight. Unfortunately, according to our findings, magnesium’s efficiency was quite modest, since most of the participants did not report a significant change in their migraine symptom control.

There are several limitations which might be ascribed to this study. All of our subjects belong to the Serbian population, and, therefore, different inherited dispositions and specific demographic characteristic that exist in different populations cannot be neglected. The study sample might be considered as an additional limitation, as a larger study sample might provide better insight into risk factors and predictors of adverse maternal and perinatal outcomes. Self-reported questionnaires are another important limitation, and incorporating standardized migraine diaries or validated scales could improve the reliability of the results.

## 5. Conclusions

The interplay between migraines, obesity, and pregnancy represents a complex and clinically relevant area of study. Depending on the stage of pregnancy and the individual’s background, pregnancy induces substantial physiological changes that can both alleviate and exacerbate migraine symptoms. Meanwhile, obesity remains a prevalent, modifiable risk factor that not only increases the intensity and frequency of migraine symptoms, but may also compromise the pregnancy course and outcome. The co-occurrence of migraines and obesity during pregnancy may amplify health risks for the mother and fetus, including heightened susceptibility to gestational diabetes mellitus. Understanding the mechanisms linking migraines, obesity, and pregnancy, including significant hormonal shifts, metabolic dysregulation, and systemic inflammation, is essential for developing targeted interventions and reducing associated risk factors. Future research should prioritize better understanding the causal relationships between pregnancy, migraines, and obesity and providing treatment strategies that focus on weight management and the control of migraine symptoms and associated comorbidities.

## Figures and Tables

**Table 1:** Distribution of evaluated variables regarding presence of migraine during pregnancy.

Variables		No Migraine During Pregnancy	Present Migraine During Pregnancy	*p*
Parity, *n* (%)	1	57 (60.0)	124 (48.6)	0.039 *
	2	34 (35.8)	93 (36.5)	
	3	4 (4.2)	37 (14.5)	
	4	0 (0.0)	1 (0.4)	
BMI category, *n* (%)	<30 kg/m^2^	83 (87.4)	167 (65.5)	<0.001 **
	>30 kg/m^2^	12 (12.6)	88 (34.5)	
HDP, *n* (%)	No	89 (93.7)	223 (87.5)	0.122 **
	Yes	6 (6.3)	32 (12.5)	
GDM, *n* (%)	No	86 (90.5)	177 (69.4)	<0.001 *
	Group 1	3 (3.2)	8 (3.1)	
	Group 2	6 (6.3)	70 (27.5)	
Thyroid gland disfunction, *n* (%)	No	75 (78.9)	193 (75.7)	0.572 **
	Yes	20 (21.1)	62 (24.3)	
Age of the migraine onset, MV ± SD		16.56 ± 5.85	15.18 ± 3.16	0.348 ***
Migraine associated with MC, *n* (%)	No	70 (73.7)	162 (63.5)	0.202 *
	Yes	2 (2.1)	8 (3.1)	
Migraine associated with stress/sleep deprivation/time change, *n* (%)	No	62 (65.3)	160 (62.7)	0.959 *
	Stress	11 (11.6)	34 (13.3)	
	Sleep deprivation	12 (12.6)	35 (13.7)	
	Whether change	10 (10.5)	26 (10.2)	
Positive family history for migraine, *n* (%)	No	58 (61.1)	156 (61.2)	0.983 *
	Yes	37 (38.9)	99 (38.8)	
Prematurity, *n* (%)	No	91 (95.8)	248 (97.3)	0.498 **
	Yes	4 (4.2)	7 (2.7)	
NICU admission, *n* (%)	No	2 (2.1)	1 (0.4)	0.180 **
	Yes	93 (97.9)	254 (99.6)	
IUGR, *n* (%)	No	92 (96.8)	247 (96.9)	0.992 *
	Yes	3 (3.2)	8 (3.1)	
Fetal Macrosomia, *n* (%)	No	89 (93.7)	239 (93.7)	0.989 *
	Yes	6 (6.3)	16 (6.3)	
Delivery mode, *n* (%)	Vaginal	82 (86.3)	218 (85.5)	0.844 *
	Cesarian section	12 (12.6)	32 (12.5)	
	Instrumental	1 (1.1)	5 (2.0)	

BMI—body mass index; HDP—hypertensive disorder of pregnancy; GDM—gestational diabetes mellitus; NICU—neonatal intensive care unit; MC—menstrual cycle; IUGR—intrauterine growth restriction; *—Chi squared test; **—Fisher’s exact test; ***—Mann–Whitney U test.

**Table 2:** Distribution of evaluated variables regarding BMI category during pregnancy.

Variables		BMI		*p*
		<30 kg/m^2^	>30 kg/m^2^	
HDP, *n* (%)	No	231 (92.4)	81 (81.0)	0.002 *
	Yes	19 (7.6)	19 (19.0)	
GDM, *n* (%)	No	207 (82.8)	56 (56.0)	<0.001 *
	Group 1	9 (3.6)	2 (2.0)	
	Group 2	34 (13.6)	42 (42.0)	
IUGR, *n* (%)	No	241 (96.4)	98 (98.0)	0.735 **
	Yes	9 (3.6)	2 (2.0)	
Fetal microsomia, *n* (%)	No	234 (93.6)	94 (94.0)	0.889 *
	Yes	16 (6.4)	6 (6.0)	
Migraine pain intensity during pregnancy versus pre-pregnancy, *n* (%)	None before pregnancy	4 (1.6)	4 (4)	<0.001 *
	None in pregnancy	79 (31.6)	8 (8.0)	
	Mild intensity	82 (32.8)	40 (40.0)	
	Same intensity	43 (17.2)	30 (30.0)	
	Severe intensity	42 (16.8)	18 (18.0)	
Migraine frequency during pregnancy versus pre-pregnancy, *n* (%)	None before pregnancy	3 (1.2)	3 (3.0)	<0.001 *
	None in pregnancy	79 (31.6)	8 (8.0)	
	Lower frequency	108 (43.2)	55 (55.0)	
	Same frequency	22 (8.8)	3 (3.0)	
	Increased frequency	38 (15.2)	31 (31.0)	
Migraine pain intensity postpartum versus during pregnancy, *n* (%)	No migraine symptoms	56 (22.4)	12 (12.0)	0.054 *
	Mild intensity	31 (12.4)	21 (21.0)	
	Same intensity	157 (62.8)	64 (64.0)	
	Severe intensity	6 (2.4)	3 (3.0)	
Migraine frequency postpartum versus during pregnancy, *n* (%)	No migraine symptoms	56 (22.4)	12 (12.0)	0.146 *
	Lower frequency	48 (19.2)	25 (25.0)	
	Same frequency	139 (55.6)	60 (60.0)	
	Increased frequency	7 (2.8)	3 (3.0)	
Migraine frequency, MV ± SD	First trimester	1.85 ± 4.12	2.30 ± 2.49	0.001 ***
	Second trimester	1.44 ± 2.96	1.28 ± 1.94	0.196 ***
	Third trimester	0.29 ± 1.48	0.73 ± 2.67	0.004 ***
Mg treatment, *n* (%)	Yes	71 (28.4)	56 (56.0)	<0.001 *
	No	179 (71.6)	44 (44.0)	
Results following Mg treatment, *n* (%)	Did not use	179 (71.6)	44 (44.0)	<0.001 *
	No change	23 (9.2)	31 (31.0)	
	Symptoms resolved quickly	48 (19.2)	25 (25.0)	

HDP—hypertensive disorder of pregnancy; GDM—gestational diabetes mellitus; IUGR—intrauterine growth restriction; Mg—magnesium; MV—mean value; SD—standard deviation; *—Chi squared test; **—Fisher’s exact test; ***—Mann–Whitney U test.

**Table 3:** Multiple logistic regression of tested variables regarding presence of migraine during pregnancy.

Variables	Multiple Logistic Regression		
	OR	95% (CI)	*p*
Parity	1.766	1.190–2.619	0.005
BMI category	2.691	1.347–5.376	0.005
Age of the migraine onset	0.918	0.865–0.974	0.004
GDM			0.004
GDM Group 1	1.292	0.319–5.226	0.720
GDM Group 2	4.788	1.915–11.970	0.001

BMI—body mass index; GDM—gestational diabetes mellitus; OR—odds ratio; CI—confidence interval.

## Data Availability

Study data are available upon reasonable request from the first author.
